# Myco-fabricated silver nanoparticle by novel soil fungi from Saudi Arabian desert and antimicrobial mechanism

**DOI:** 10.1038/s41598-024-63117-5

**Published:** 2024-07-02

**Authors:** Afrah E. Mohammed, Shereen M. Korany, Hana Sonbol, Eman A. Alhomaidi, Suaad S. Alwakeel, Reham M. Elbaz

**Affiliations:** 1 Department of Biology, College of Science, Princess Nourah bint Abdulrahman University, P.O. Box 84428, 11671 Riyadh, Saudi Arabia; 2 Microbiology and Immunology Unit, Natural and Health Sciences Research Center, Princess Nourah bint Abdulrahman University, P.O. Box 84428, 11671 Riyadh, Saudi Arabia; 3 Botany and Microbiology Department, Faculty of Science, Helwan University, Cairo, 12612 Egypt; 4 Department of Biology, College of Science, University of Bisha, P.O. Box 551, 61922 Bisha, Saudi Arabia

**Keywords:** Nanostructure, Soil fungi, *Embellisia* spp., *Gymnoascus* spp., Ultrastructural changes, Biological techniques, Nanobiotechnology, Nanoparticles, Antimicrobial resistance, Microbiology, Fungi, Fungal biology

## Abstract

Biological agents are getting a noticeable concern as efficient eco-friendly method for nanoparticle fabrication, from which fungi considered promising agents in this field. In the current study, two fungal species (*Embellisia* spp. and *Gymnoascus* spp.) were isolated from the desert soil in Saudi Arabia and identified using 18S rRNA gene sequencing then used as bio-mediator for the fabrication of silver nanoparticles (AgNPs). Myco-synthesized AgNPs were characterized using UV–visible spectrometry, transmission electron microscopy, Fourier transform infrared spectroscopy and dynamic light scattering techniques. Their antibacterial activity against *Escherichia coli, Pseudomonas aeruginosa, Staphylococcus aureus,* and *Klebsiella pneumoniae* were investigated. In atrial to detect their possible antibacterial mechanism, Sodium dodecyl sulfate (SDS-PAGE) and TEM analysis were performed for *Klebsiella pneumoniae* treated by the myco-synthesized AgNPs. Detected properties of the fabricated materials indicated the ability of both tested fungal strains in successful fabrication of AgNPs having same range of mean size diameters and varied PDI. The efficiency of *Embellisia* spp. in providing AgNPs with higher antibacterial activity compared to *Gymnoascus* spp. was reported however, both indicated antibacterial efficacy. Variations in the protein profile of *K. pneumoniae* after treatments and ultrastructural changes were observed. Current outcomes suggested applying of fungi as direct, simple and sustainable approach in providing efficient AgNPs.

## Introduction

A great concern has been given to the potential uses of the metal nanoparticles (MN) that have size in the range 1–100 nm, due to their significant optical^1^, physical^2^, and chemical^3^ characteristics in relation to their bulk origin. Such unique properties facilitated their applications in various fields of science and technology. In the medical and food sectors, for both biosensing and microbial detection, MN could be ideal because of their biocompatibility, ability in electron transfer, and reaction catalysis^4^, beside other applications in diagnosis and therapy. MN could rapidly detect the microbes and tumor cells disease-related biomarkers, however, they also demonstrate capability for single-cell evaluation^5^.

Biomedical and biotechnological applications are the cornerstone for MN usage. Some of their important biomedical applications, but not limited to, are anti-inflammatory, antioxidant, antibacterial, antifungal, cytotoxic, drug delivery and detecting agents^6,7^. However, bioremediation, biodeterioration and wastewater treatment are their most known biotechnological applications^8,9^. Among the MN, silver nanoparticles (AgNPs) attracted a great interest due to their chemical, physical and biological characteristics^10,11^. AgNPs have high ability against microbes, and cancer cells^12^ besides, they have been used also in wound dressings, diagnostics, catalysis, biosensors and drug delivery^13–15^. Alongside the aforementioned biomedical applications, AgNPs can be applied in different biotechnology lines, such as food preservation and water filtration, in addition to spray and sanitization^13,16^.

With the emergence of antimicrobial resistance, the need for alternative antimicrobial agents to replace the conventional ones enhanced researchers to develop AgNPs as one of the good options for microbial mitigation. Previous studies have approved the ability of AgNPs as antimicrobial agents with significant effects against some multidrug- resistant bacteria^12,17,18^. The unique and wide range of AgNPs applications are the factors that led to the development of various fabrication methods for efficient and safe usage^19–21^.

Although chemical and physical methods are the best ways to develop nanoparticles with unique size and shape in a short time, however, their weakness points are the high cost and the production of hazardous by-products^22,23^. Therefore, the green route could be a good alternative because it is an easy, applicable, and eco-friendly approach. Such route includes using microbes and plants, beside other biomolecules as biogenic agents^14,16^.

Stable nanoparticles with better distributed size and morphology were noted from the biogenic approach in relation to those produced by other methods^24^. The molecules from biogenic agents that may cap the MN enhance their stability and dispersity^25^. Generally, nanoparticles produced with the aid of biological routes are harmless, environmentally benign that can be used in agriculture, medicine, biochemical detection and related fields. On this regard, the use of microbes as one approach of the biogenic synthesis is gaining great interest. From which fungi can be used for AgNPs development by extra or intracellular approaches however, the extracellular processis easier and faster than the intracellular one^26^. Previous studies demonstrated extracellular approach for AgNPs fabrication using fungi^18,25^ Employment of fungi is an important fabrication approach due to their ability to produce greater amounts of proteins in relation bacteria^27^ and their high intracellular metal uptake and binding capacity^28^. In this respect, it is expected that, fungi from extreme conditions might have unique compounds that enhance their ability to withstand harsh environments, but also could help efficiently in AgNPs production. Therefore, the current study was designed to provide new candidates in AgNPs fabrication as safe approach in providing high stable and active materials. Two fungal species (*Embellisia* spp. and *Gymnoascus* spp.) isolated from the desert soils in Saudi Arabia, not addressed before, were selected. Hence, present study represents the first report on these two species as biogenic agents in extracellular AgNPs fabrication using fungal filtrate as source of their biomolecules. Thereafter, the fabricated AgNPs were characterized using UV, DLS, TEM, SEM, and FTIR and tested against some bacterial pathogenic strains. In a trial to detect the possible antibacterial mechanism of the myco-fabricated AgNPs, treated bacteria were investigated using TEM and SDS-PAGE analysis.

## Materials and methods

### Materials

The laboratory experiments have been done at the faculty of science laboratory, Princess Nourah bint Abdulrahman University, Riyadh, Saudi Arabia. Where the silver nitrate, potato-dextrose agar (PDA), Czapek-Dox Agar with 1% chloramphenicol, nutrient agar and broth and Sabouraud broth (Oxoid, UK) were obtained. The soil fungi (*Embellisia* spp. and *Gymnoascus* spp.) have been isolated from Saudi Arabian desert soils. Protein profile and TEM analysis were done at king Saud university laboratory.

### Isolation of fungi

Two fungal strains (*Embellisia* spp. and *Gymnoascus* spp.) were isolated from ALkharj and ALQasab respectively, Saudi Arabian desert soils. The soil was collected from 5 to 20 cm depth, then subjected to culture following the dilution plate method, as described elsewhere^29^. Two culture media were used: PDA and Czapek-Dox Agar with 1% chloramphenicol. The plates were incubated for 7 days at 28 °C. Pure fungal colonies were kept at 4 °C for further analysis.

### Molecular identification using 18S rRNA gene.

A PCR assay for partial amplification of the fungal 18S rRNA genes was performed as described previously^30^ with the primers NS1 F (5′ GTAGTCATATGCTTGTCTC 3′) and NS8 R (5′ TCCGCAGGTTCACCTACGGA 3′). The PCR reaction mixture contained 2 µL of 10 × PCR Buffer, 1.6 µL of 2.5 mM dNTPs, 1.0 µL of 10 pmol/µL from each primer, 0.2 µL of KOMA Taq (2.5 U/µL), 2 µL of 20 ng/µL DNA template and HPLC-grade water to adjust the reaction volume to 20 µL. The amplification reactions were done in a Biometra thermal cycler (Analytik Jena, Jena, Germany) with the reaction conditions mentioned elsewhere^31^. Confirmation of successful amplification was assured by applying the PCR products to agarose gel electrophoresis. The PCR products were purified by PCR Cleanup Kit (MilliporeSigma, Burlington, MA, USA).

#### Sequence analysis

The purified PCR products were sent to a commercial company (Marogen Europe, Amsterdam, The Netherlands) for partial sequencing of the 18S rDNA using the same amplification primers. Sequencing at the company was performed by the BigDye Terminator v3.1 Cycle Sequencing Kit (Applied Biosystems, USA) and 3730xl DNA Analyzer automated DNA sequencing system (Applied Biosystems). The retrieved 18S rDNA sequences of the two fungal isolates were analyzed using the software Geneious Prime Version 2020.1.2^32^. Consent sequences were produced from forward and reverse sequences. The edited sequences were compared with other sequences of related reference strains in nucleotide database of the National Center of Biotechnology Information (NCBI) of the United States using the nucleotide platform of the Basic Local Alignment Search Tool (BLASTn). The sequences were also used to construct a phylogenetic tree using the Neighbor-Joining method^33^ with the software MEGA X^34^.

### Fungal culture filtrate

Separate culture flasks containing 500 mL of Sabouraud broth (Oxoid, UK) were inoculated by each fungal species isolate individually. The flasks were incubated in static incubator for 7 days at 28 °C, after which the fungal biomass was collected by filtration through Whatman filter paper No. 1 and washed with sterile distilled water to remove excess medium. The fungal biomasses were weighed, and 0.5 g were added to distilled water (10 mL) and incubated at 28 °C for 72 h. Finally, the biomass was separated from the filtrate by filtration. The aqueous filtrate containing the fungal metabolites was kept for further usage in the refrigerator at 4 °C^35^.

### Myco-synthesis of AgNPs

For the biosynthesis of AgNPs, 1 mM AgNO_3_ was added to fungal aqueous filtrate at a ratio 1:1 and then boiled for 30 min. This mixture was incubated under natural sun light at 25 °C for 24 h, till a dark brown color was formed. The nanoparticles were separated by centrifugation of the mixture at 14,000 rpm for 15 min, then dried by dropping the precipitate on petri dishes at room temperature and kept for further analysis^36^.

### Characterization of myco-synthesized AgNPs

AgNPs was re-dispersed in 1 mL of distilled water for chracterization. A UV–visible spectrophotometer (BIOCHROM Libra S60PC, England) was used in the range of 200–600 nm for measuring the absorbance of the AgNPs with the fungal aqueous filtrate was used as blank, as described before^35^. The morphology, average diameter and distribution were estimated by transmission electron microscopy, TEM (JEM-1011, JEOL, Tokyo, Japan) at 80 kV voltage. The pattern of the size distribution (size and poly dispersed index, PDI) and zeta potential were assessed by a dynamic light scattering (DLS) system by a Zetasizer (NANO ZSP, Malvern Instruments Ltd., SerialNumber: MAL1118778, version 7.11, Malvern, UK). For surface analysis of the synthesized NPs, a scanning electron microscope, SEM (JED-2200 series, JEOL) provided by energy-dispersive X-ray spectroscopy (EDX) was used to confirm the presence of elemental silver, as previously described^37^. Functional groups of fungal aqueous filtrates responsible for AgNO_3_ reduction were estimated with Fourier transform infrared (FT-IR) spectroscopy (SPECTRUM100, Perkin-Elmer, Wellesley, MA, USA) with a range 450–3500 cm^−1^, as detailed earlier^38^.

### Investigation of the antibacterial activity of myco-synthesized AgNPs

To assess the antibacterial potential of the myco-synthesized AgNPs in vitro, four human pathogenic bacteria were tested (*Escherichia coli, Pseudomonas aeruginosa Staphylococcus aureus,* and *Klebsiella pneumoniae*). The assay was conducted by the agar well diffusion technique^39^. Using sterile swabs, 100 µL of 24 h. old bacterial culture with a concentration equal to 0.5 McFarland (1.5 × 10^8^ CFU mL^−1^) from each of the four tested strains were streaked on a plate of nutrient agar and left to dry. Under aseptic conditions, wells of 0.8 mm in diameter were made on the agar using a sterile cork-borer. Volumes of 50 µL of myco-synthesized AgNPs were added to the wells. The fungal filtrate and 5 µg ampicillin discs were used as negative and positive controls, respectively. The plates were incubated at 37 °C for 24 h. The experiment was performed in triplicate and the growth inhibition zones were estimated in millimeters (means ± standard variation), as described previously^40^.

The minimum inhibitory concentration (MIC) and the minimum bactericidal concentration (MBC) were estimated using the microdilution method in nutrient broth. The bacterial cultures of the four tested strains were individually diluted to 0.5 McFarland from which a volume of 10 µL was added to nutrient broth (350 µL). The isolates were tested against the myco-fabricated AgNPs solubilized in Dimethyl sulfoxide (DMSO) at different concentrations (0.031, 0.062, 0.125 and 0.250 mg mL^−1^). The negative control composed of nutrient broth inoculated with bacterial inoculum. Then, the plates were incubated at 37 °C for 24 h. The MIC was determined as the lowest concentration that inhibit observable bacterial growth^41^ where the MBC was verified when no growth appeared when treated bacteria were sub-cultured on nutrient agar plates after incubation period^42^.

### Protein profile pattern by SDS-PAGE analysis

The total cellular soluble proteins of *K. pneumoniae* were extracted before and after treatment with 1 mg mL^−1^ of AgNPs synthesized from each fungal strain for 24 h at 37 °C, then purified by TriFast (Peqlab, VWR company). The proteins were further fractionated by OmniPAGE Mini vertical electrophoresis unit provided with a power Pro 5 power supply (Cleaver Scientific, Warwickshire, UK) on a SERVAGel™ TG PRiME 10% (SERVA, Heidelberg, Germany)^43^.

### Detection of ultrastructural changes in AgNPs treated bacteria

One species (*K. pneumoniae*) was chosen for TEM analysis (JEM-1011, JEOL, Tokyo, Japan, at 80 kV voltage) to test the possible structural changes for cells treated with AgNPs fabricated by both fungal strains and incubated for 24 h. at 37 °C. Thereafter, bacterial suspension was centrifuged at 4000 rpm for 15 min. Then, 2.5% glutaraldehyde in 100 mM phosphate buffer, pH 7.0, was added to the pellet for sample fixation for 15–30 min. This was followed by applying 1% osmium tetroxide in 100 mM phosphate buffer for 1–2 h. at 4 °C, then washed five times with distilled water. Then, En bloc stain with 2% aqueous uranyl acetate was applied for ~ 2 h at 4 °C in the dark. Dehydration was done with series of acetone (30, 50, 70, 90 and 100%). After that, the samples were infiltrated in propylene oxide, followed by embedding in fresh resin (Epon mixture) for 12–24 h. at 60–70 °C. The ultrathin sections of the samples were put on 200-mesh copper grids after double staining with 2% uranyl acetate and lead citrate, then examined under a JEOL 100 CX electron microscope operating at 80 kV, as described previously^44^.

### Statistical analysis

All data were represented as mean and standard deviations. One-way analysis of variance (ANOVA) as well as graphs preparation were performed by Graph-bad Prism 9.1 software (Inc., La Jolla, CA, USA). ImagJ software was used for calculating the particles size and diameter from TEM images.

## Results

### Fungal strains isolation and identification

**Figure 1:**
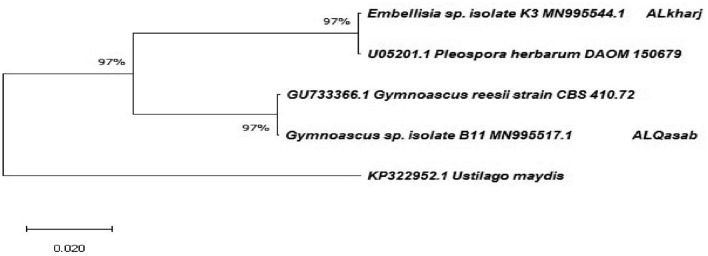
Phylogenetic tree of fungal isolates based on 18S rDNA constructed using the Neighbor-Joining method in MEGA X. The accession number shown at the end of the branch and their closest relatives’ sequences from the GenBank accession number shown at the tip of the branch. *Ustilago maydis* accession number KP322952 was an outlier. The percentage of replicate trees in which the associated taxa clustered together are shown next to the branches.

The 18S rRNA gene sequences analysis identified the two soil fungal isolates from Saudi Arabian desert as *Embellisia* spp. and *Gymnoascus* spp. The phylogenetic tree constructed from the two sequences together with corresponding to other strains worldwide is shown in Fig. 1. The sequences of these isolates have been deposited in the nucleotide sequence database of the GenBank of NCBI under accession numbers MN995544 for *Embellisia* spp. and MN995517 for *Gymnoascus* spp.

### Myco-synthesis of AgNPs

The individual mixture of the aqueous filtrates from both fungal strains and the AgNO_3_ has been changed from colorless to a stable brown color after incubation for 24 h. Such changes indicated the reduction of silver ions to AgNPs by fungal secondary metabolites. This conversion was the first sign that *Embellisia* spp. and *Gymnoascus* spp. were capable in AgNPs fabrication from AgNO_3_ providing E-AgNPs and G-AgNPs, respectively.

### Myco-synthesized AgNPs characterization

**Figure 2:**
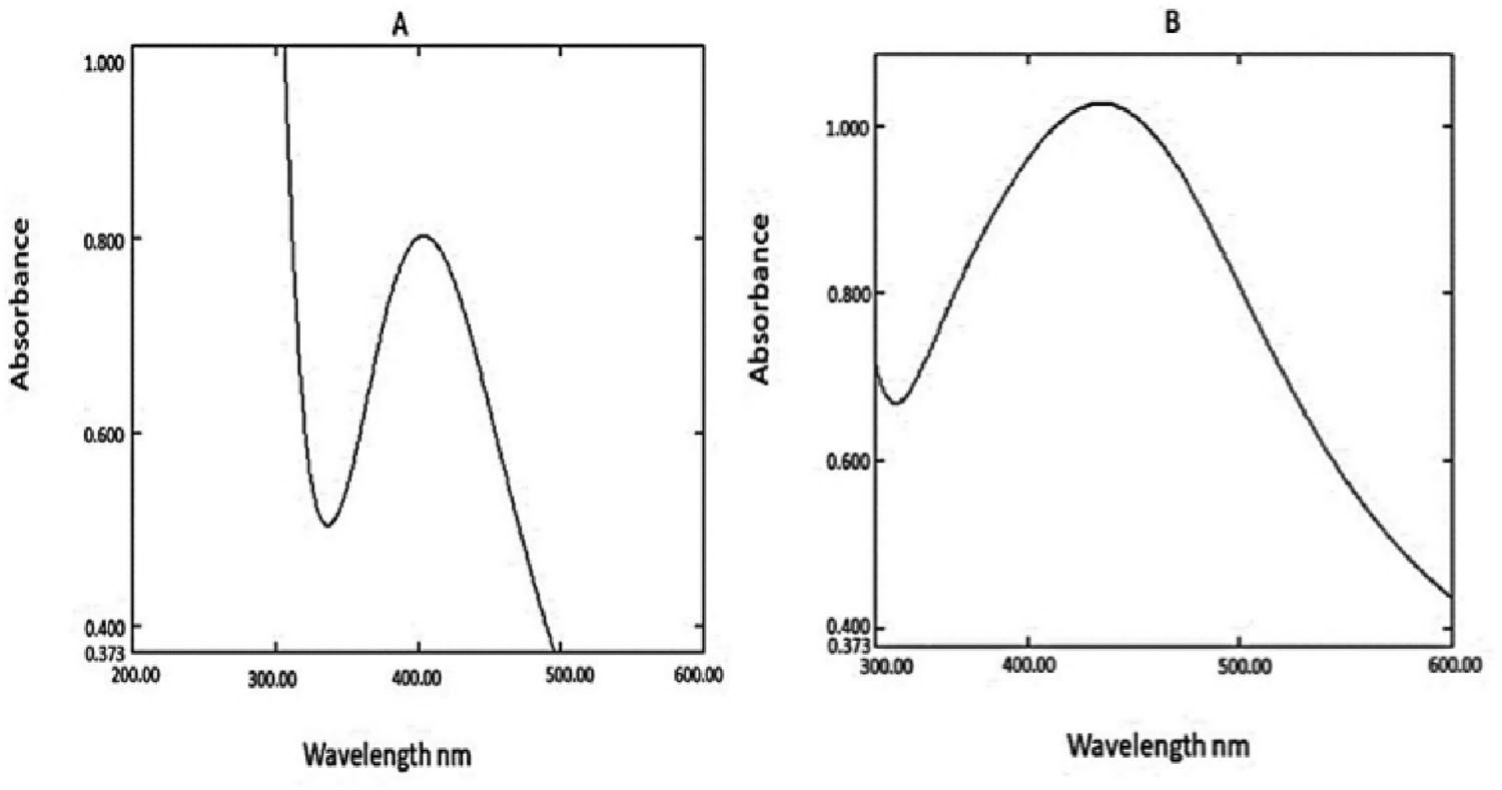
UV–visible absorption spectrum of E-AgNPs (**A**) and G-AgNPs (**B**) synthesized using *Embellisia* spp. and *Gymnoascus* spp., respectively.

UV–visible spectrophotometry was used to further confirmation of the AgNPs synthesis from fungal filtrate. Strong beaks at 430 and 450 nm for E-AgNPs and G-AgNPs, respectively, were reported (Fig. 2A,B). The absorption peaks were detected in relation to the fungal filtrate (blank).

**Figure 3:**
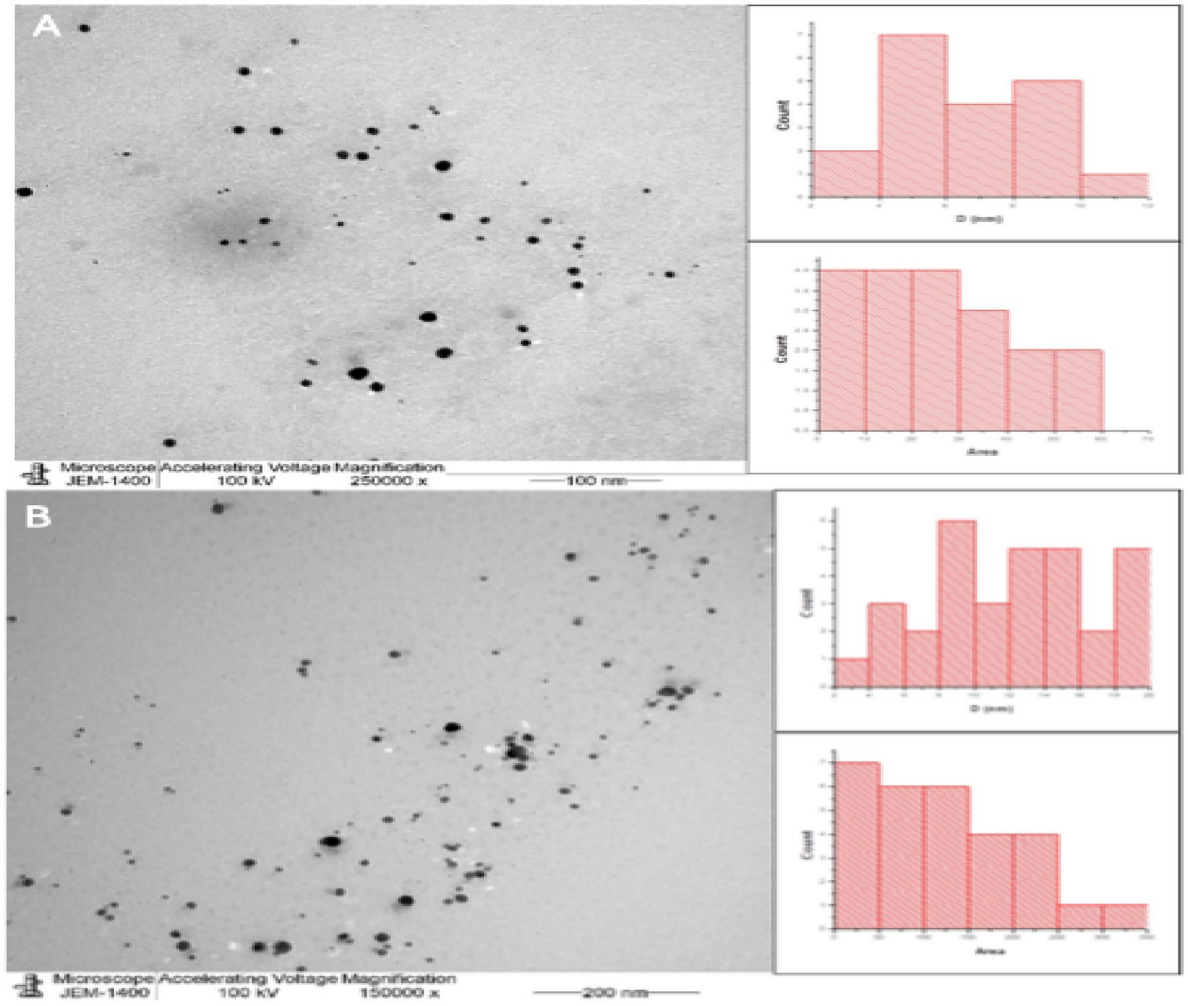
TEM micrograph showing morphological characterization of myco-synthesized using *Embellisia* spp (E-AgNPs, (**A**) and *Gymnoascus* spp., (G-AgNPs, **B**) and at magnifications of 250,000 and 150,000, respectively. Bar figures indicating the particles distribution (diameter and area) that calculated using imagJ software.

The morphology and the size diameter of the myco-synthesized E-AgNPs and G-AgNPs were observed using TEM analysis which indicates well-dispersed spherical nanoparticles of 2–20 nm size diameter (Fig. 3A,B).

**Figure 4:**
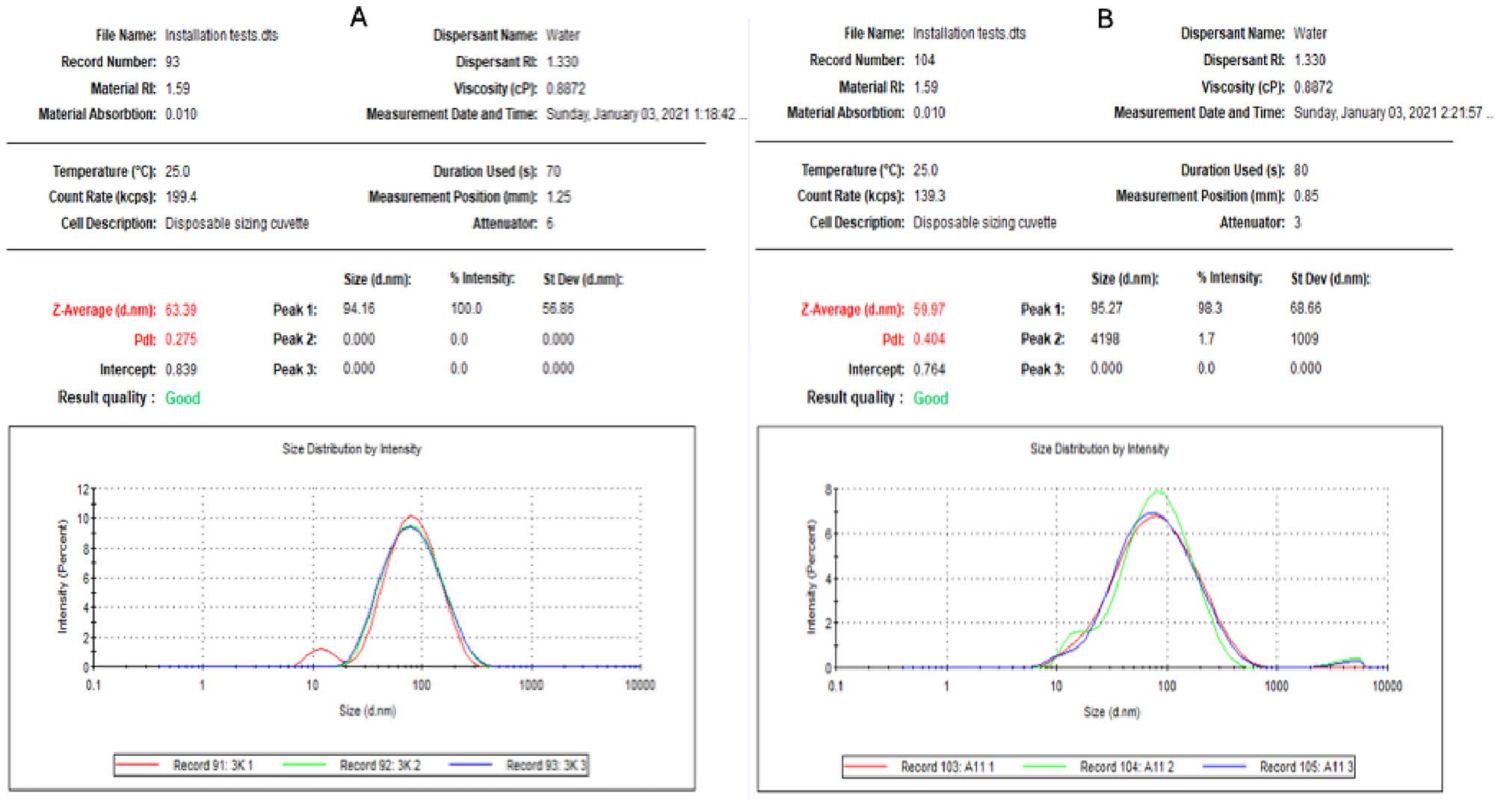
Size distribution of myco-synthesized E-AgNPs (**A**) and G-AgNPs (**B**) prepared using *Embellisia* spp. and *Gymnoascus* spp., respectively. The average diameter was calculated from three reading.

Further, analysis was done by DLS, that determined an average diameter of 63.39 nm for E-AgNPs and 59.97 nm for G-AgNPs with a polydispersity index (PDI) of 0.28 and 0.40, respectively (Fig. 4).

**Figure 5:**
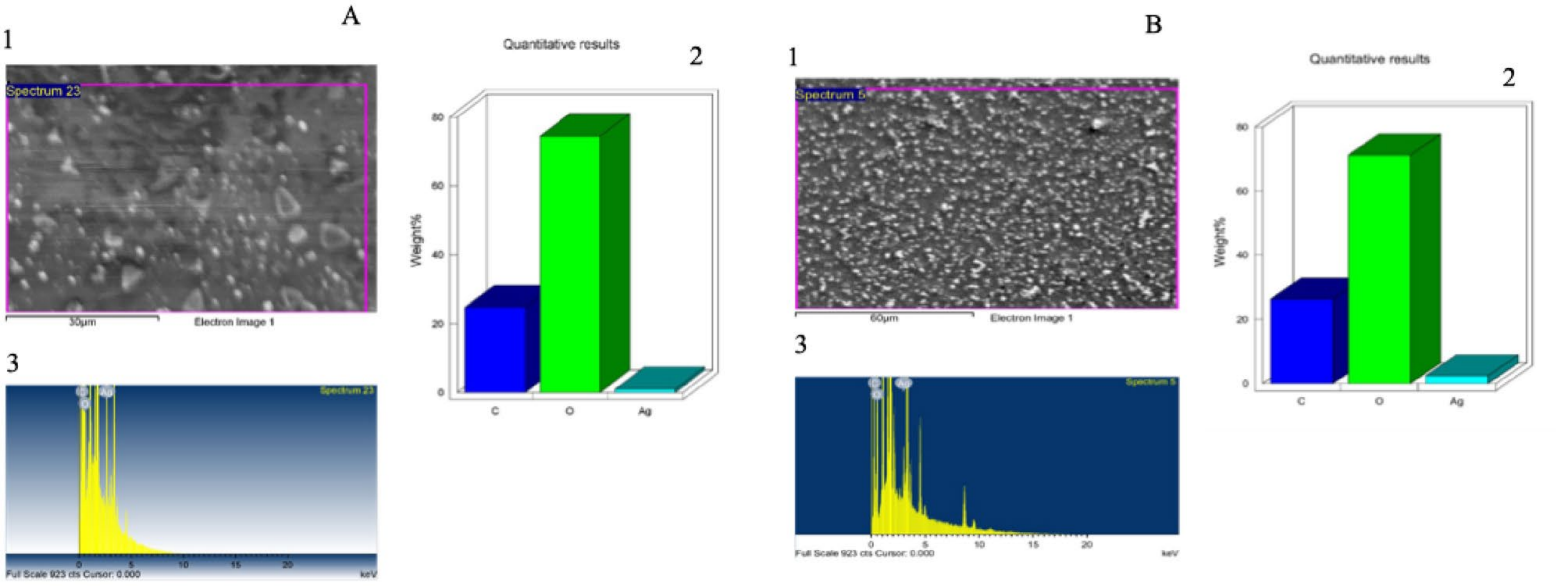
Surface morphology of E-AgNPs prepared by *Embellisia* spp (**A**) and G-AgNPs by *Gymnoascus* spp. (**B**). SEM images (1) indicating particle morphology, quantitative data analysis of images that presenting the relative composition of carbon, oxygen, and silver atoms (2) and EDS spectrum (3).

EDX analysis confirmed the presence of the silver element in all myco-synthesized AgNPs. Strong signals at 3 keV were noted for Ag, along with carbon and oxygen peaks (Fig. 5).

**Figure 6:**
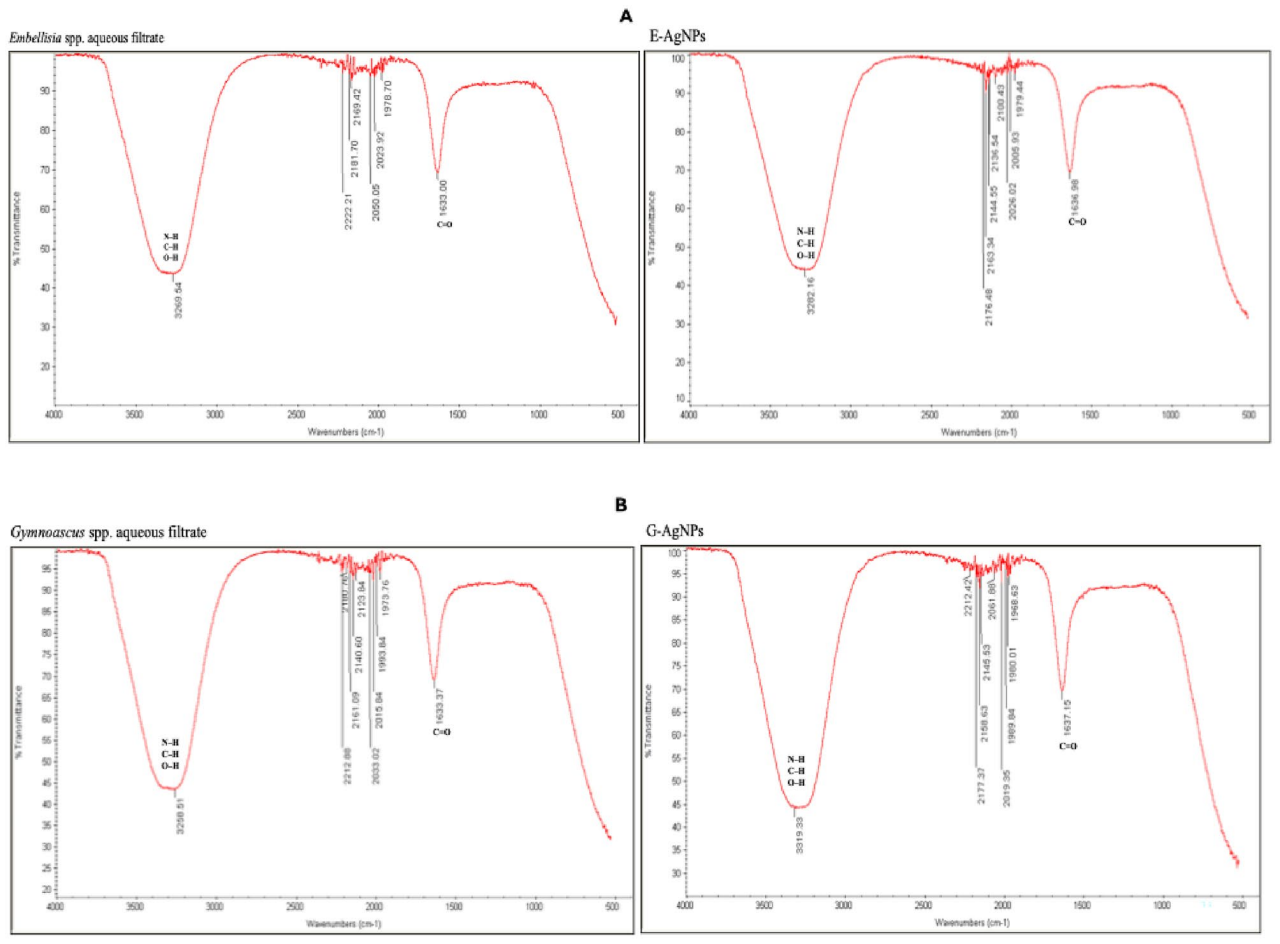
FTIR pattern for *Embellisia* spp. filtrate and the bio-synthesized E-AgNPs (**A**) beside *Gymnoascus* spp. filtrate and the bio-synthesized G-AgNPs (**B**).

The FTIR measurements were used to determine the presence of various functional groups in the fungal metabolites involved in AgNPs synthesis, capping, stabilization and consistency. The FTIR for the aqueous filtrate from *Embellisia* spp. showed eight peaks ranging from 3269.54 to 1633 cm^−1^; the peaks increased to ten for E-AgNPs ranging between 3282.16 and 1636.98 cm^−1^ (Fig. 6A). In *Gymnoascus* spp. filtrate, eleven peaks were detected ranging from 3258.51 to 1633.37 cm^−1^, whereas eleven peaks were detected for G-AgNPs ranging between 3319.33 and 1637.15 cm^−1^ (Fig. 6B).

### Antibacterial activity of the myco-synthesized AgNPs

**Figure 7:**
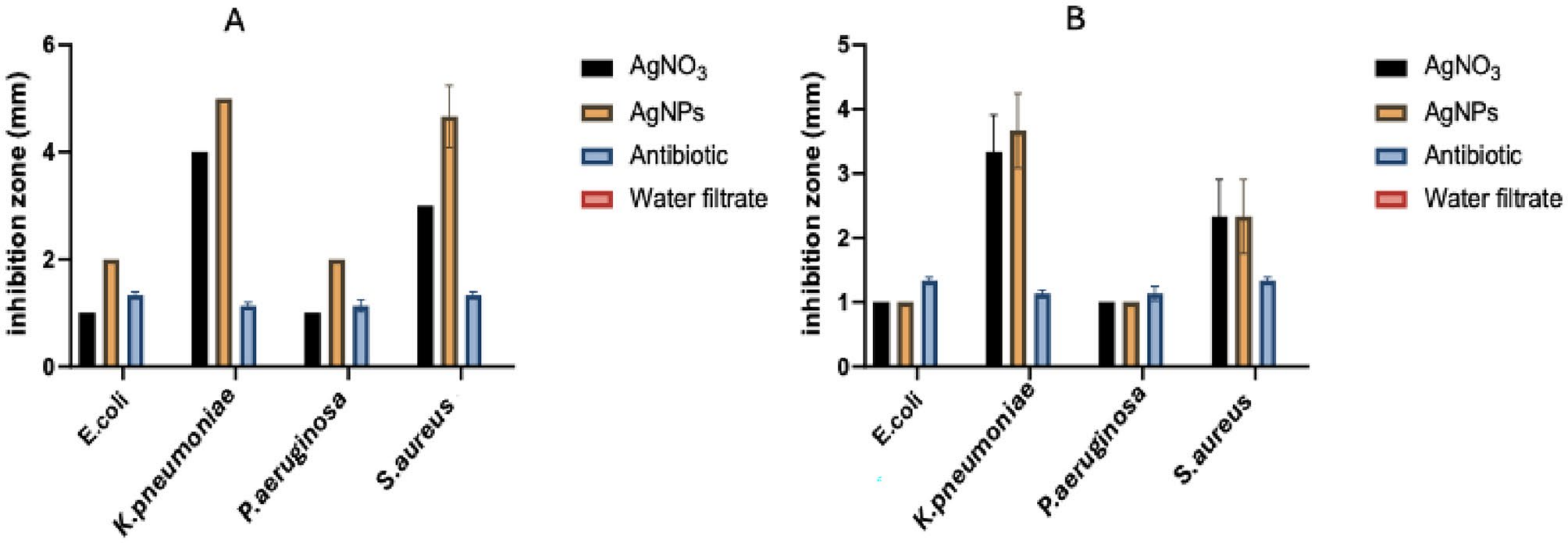
Antibacterial activity of myco-synthesized E-AgNPs (**A**) and G-AgNPs (**B**), using *Embellisia* spp. and *Gymnoascus* spp., respectively, in comparison to AgNO_3_, ampicillin antibiotic and fungal water filtrate.

When the E-AgNPs and G-AgNPs at concentrations of 1 mg mL^−1^ each were tested for antimicrobial activity against four human pathogenic bacteria. The maximum activity was recorded against *K. pneumoniae*, with growth inhibition zones of 5 and 3.8 mm for E-AgNPs and G-AgNPs, respectively. *S. aureus* was also sensitive to AgNPs with growth inhibition zones of 4.8 mm for E-AgNPs and 2.5 mm for G-AgNPs (Fig. 7A,B). The lowest activity of both AgNPs was noted against *E. coil* and *P. aeruginosa*. The activity of E-AgNPs was significantly higher than G-AgNPs (p < 0.001).Significant varitaion in the bacterial response to the myco-fabricated AgNPs was noted as well as their interaction.

The MIC and MBC estimated for both myco-synthesized AgNPs by the micro-dilution method are shown in Table 1. The lowest MIC was recorded at 0.03 μg mL^−1^ against *K. pneumoniae* for both E-AgNPs and G-AgNPs, while the lowest MBC for both AgNPs types was 0.06 μg mL^−1^ against the same strain (Table 1).

**Table 1:** MICs and MBCs for the myco-synthesized AgNPs against four pathogenic bacterial strains.

Bacterial strain	MIC (μg mL^−1^)		MBC (μg mL^−1^)	
	E-AgNPs	G-AgNPs	E-AgNPs	G-AgNPs
*E. coli*	0.06	0.06	0.12	0.12
*Klebsiella pneumoniae*	0.03	0.03	0.06	0.06
*Pseudomonas aeruginosa*	0.06	0.12	0.12	0.24
*Staphylococcus aureus*	0.06	0.06	0.12	0.12

### SDS-PAGE and protein profile pattern

**Figure 8:**
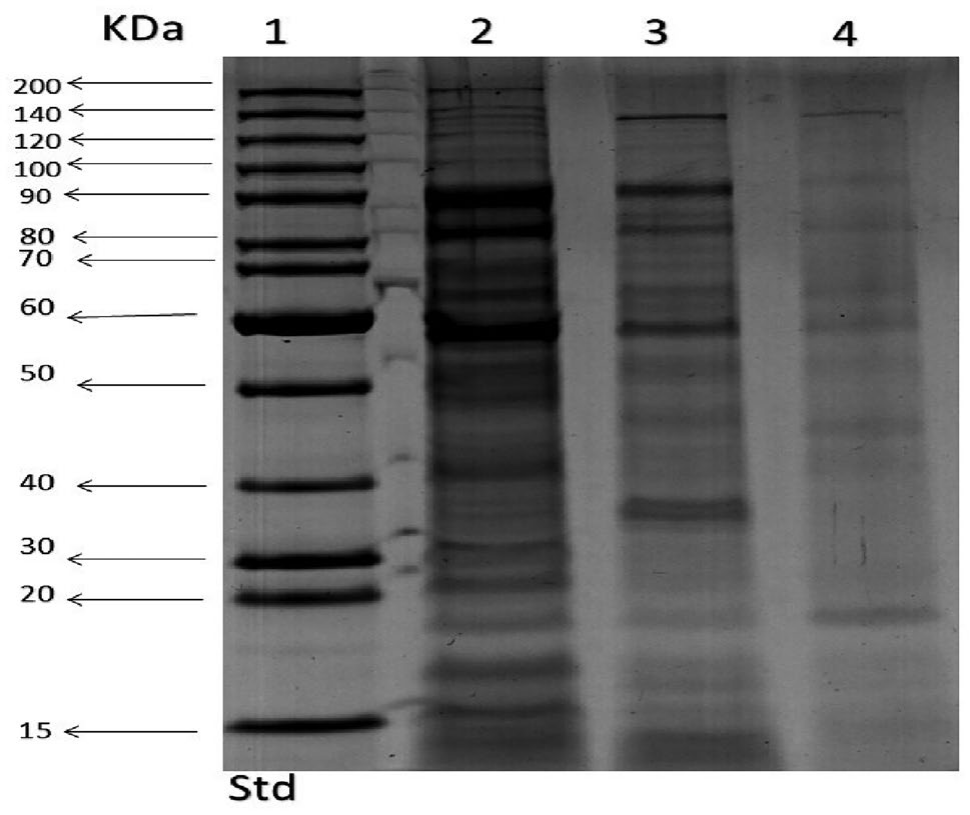
SDS-PAGE analysis and profile of protein extracted from *K. pneumoniae,* where control is indicated in lane 2 and treated bacteria with E-AgNPs and G-AgNPs synthesized using *Embellisia* spp. and *Gymnoascus* spp. are presented in lanes 3 and 4, respectively. Std refers to the molecular mass standard.

Since the highest activities for E-AgNPs and G-AgNPs were noted against *K. pneumoniae*, therefore this strain was chosen for SDS-PAGE analysis to detect the protein profiling pattern before and after AgNPs treatment. The protein pattern from treated *K. pneumoniae* (Fig. 8) displayed lower protein bands intensities as clear in lanes 3 and 4 compared with the untreated control (lane 2). The total number of protein bands of the control was 22 at sizes ranged from 15 to 160 kDa, whereas in E-AgNPs treated cells (lane 3) they were 16 bands with sizes ranged from 15 to 120 kDa. In lane 4, the protein from G-AgNPs treated bacteria showed only 8 bands ranging from 19 to 120 kDa.

### Ultrastructural changes detected in AgNPs treated bacteria

**Figure 9:**
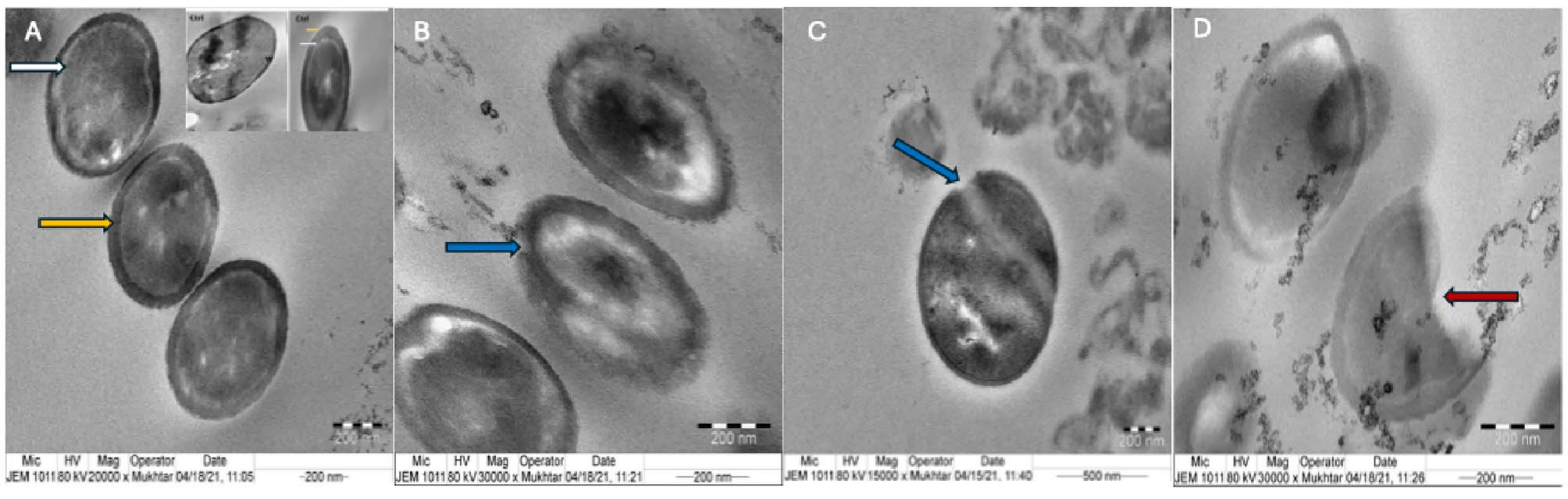
TEM micrographs of AgNPs-treated *K. pneumoniae* where untreated cells (**A**) show intact cell wall (yellow arrow) and a peptidoglycan layer in periplasmic space (white arrow). Cell treated with E-AgNPs (**B**,**C**) showed damage in the cell wall (blue arrow). G-AgNPs treated bacteria (**D**) showed cell damage (red arrow). E-AgNPs and G-AgNPs were myco-synthesized using *Embellisia* spp. and *Gymnoascus* spp., respectively. Images were taken at 80 kV and magnification of 15,000, 20,000 and 30,000×.

Examination of *K. pneumoniae* cells by TEM analysis after treatment with the myco-synthesized AgNPs (Fig. 9) showed damaged cell wall (Fig. 9B,C) and deformation and rupture of the bacterial cell (Fig. 9D).

## Discussion

It was challenging for many scientists to find out new, simplified and ecofriendly methods for obtaining efficient AgNPs. The bio-fabrications are highly recommended due to their advantages over other synthetic methods^3,37^. The utilization of fungi for the synthesis of AgNPs is remarkably useful in relation to other fabrication methods due to simple handling and harmless effect. Several strains of *Fusarium oxysporum* mediated extracellular AgNPs formation depending on reductase/electron shuttle relationship and provided NPs ranging between 20 and 50 nm^45^.

In the present work the fungal filtrates from *Embellisia* spp. and *Gymnoascus* spp. indicated their ability in reducing Ag ions for self-assembly of AgNPs as detected by the formation of dark brown color when both compounds were mixed. Additionally, the recorded absorbance peaks at the UV–visible spectrum corresponded to the typical band of AgNPs that attributed to surface plasmon resonance, confirming the production of AgNPs^46^. DLS analysis revealed that G-AgNPs particle size was smaller than that of E-AgNPs, which may reflect the diversity of fungal bioactive molecules involved in the reduction process, such as citric acid, peroxidases, homogeneous and heterogeneous proteins along with fungal enzymes, especially nitrate reductases^47^. Although the size variation was not that high however, PDI indicated better mono-dispersibility and homogeneity for E-AgNPs than G-AgNPs. TEM indicated spherical shape of NPs and EDX analysis confirmed the presence of the silver element in G-AgNPs and E-AgNPs at 3 keV, which is typical for the absorption of metallic silver nano-crystallites. The signal band detected by FTIR analysis at 3269.54 and 3282.16 cm^−1^ corresponds to N–H, C–H, and O–H stretching vibrations, indicating the presence of primary amines in the fungal proteins alkynes, and alcohols, respectively^48^. Intense absorption bands in FTIR at 1633.37 and 1637.15 cm^−1^ might be attributed to amide I due to carbonyl stretch in proteins C=O^49^. Similar functional group detected at both fungal filtrate and AgNPs fabricated by their aid indicating the role of fungal biomolecules in the reduction and capping processes of AgNPs. The biological molecules from the fungal extract may carry out a double function by performing the reduction process and enhancing the AgNPs stability, as they could serve as good capping agents that covered the AgNPs surface and prevented their aggregation^50,51^. From these biomolecules, fungal enzymes are good reducing agents^25^. Fungi could have electrostatic interactions between the metal ion and cell wall associated enzymes followed by subsequent enzymatic reduction of metal ions resulting in intracellular or extracellular production of NPs^52^.

The capability of fungal filtrates in NPs formation was previously approved for *Phoma* spp., *Chaetomium globosum*, and *Chaetomium* spp., which were able to reduce AgNO_3_ into AgNPs with varying sizes and shapes^18^. Also, *Fusarium oxysporum* and *Verticillium* spp. were capable of reducing Ag and gold into their nanoparticle forms depending on their extracellular proteins and secondary metabolites^52^.

Current findings showed potent antibacterial against the four tested human pathogenic bacteria however, the activity of E-AgNPs was higher than G-AgNPs. The reasons behind the variation in the NPs activity could be attributed to the variation in their PDI characteristics since no clear variations were noted in their size and morphology. PDI bellow 0.3 reflecting the mono dispersity and uniformity of the E-AgNPs^53^ that might enhance their activity compared to G-AgNPs where their PDI was 0.4. *K. pneumoniae* was the most sensitive tested strain, which is in accordance with previous findings by Sonbol et al.^18^, who observed strong antibacterial activity of three myco-synthesized AgNPs. The antibacterial activity of morphologically modified AgNPs and chitosan loaded essential oils, were reported against multidrug resistant Gram negative bacteria and biofilm forming *Acinetobacter baumannii,* respectively^54,55^.

The antibacterial ability of AgNPs could be related to the electrostatic interactions between positively charged Ag ions and the negatively charged bacterial membrane, which would lead to membrane permeability disruption and cell wall damage^56,57^. AgNPs could have also bound a specific group of enzymes (thiols) and affected the DNA capacity to replicate leading to cell death^58^. In this study, the MIC and MBC indicated low fungal based AgNPs concentrations were needed to suppress the growth of both tested Gram-negative and Gram-positive bacteria. The efficiency of mucogenic AgNPs as antibacterial agents could be associated with their ability to enhance cellular oxidative stress causing destruction of the cell components leading to cell death.

Polyacrylamide gel electrophoresis (SDS-PAGE) is a powerful technique for molecular analysis of cell proteins, especially in comparing protein patterns to detect qualitative, and quantitative changes, as well as clustering of bacterial groups^59^. Proteomic differences of *K. pneumoniae* before and after treatment with fungal-based AgNPs might indicate significant polymorphism in the protein profiles that could be attributed to degradation or even block of the pathways for biosynthesis of certain proteins. Lower protein band intensity compared with untreated control might also be attributed to oxidative stress enhanced by NPs treatment. Besides, unfolding of the protein chain resulting from the reaction of AgNPs with thiol groups of the proteins^60^ is expected to cause degradation of the proteins. Therefore, it might explain the reduction in the number of protein bands from 22 in untreated to 16 and 8 in the treated *bacteria* with E-AgNPs and G-AgNPs, respectively. Different studies reported that AgNPs may be involved in preventing protein synthesis by preventing many translation factors^61,62^.

Moreover, TEM imaging of *K. pneumoniae* treated with fungal-based AgNPs showed disruption within the cell membrane that led to damage in the outer membrane and deformation of the bacterial cell. As a result of interaction of free Ag^+^ with several cellular sites, such as cytoplasmic membrane, cytoplasm and nuclear matrix. So, membrane permeability increased leading to loss of certain membrane ions as K^+^ that impaired membrane integrity, and effects on the respiration process^63,64^. Other reports linked AgNPs toxicity to induce oxidative stress by motivating the development of ROS, which lead to cellular dysfunction and death^65^. In general, the unique characters of NPs, such as their smaller size with larger surface area, their charges enable them to easily attach and penetrate bacterial cell walls and membranes^44^ make them a perfect tool from biological source to control adverse antibiotic resistant human pathogens.

## Conclusion

The present investigation indicated the ability of two soil fungal isolates from Saudi Arabian desert (*Embellisia* spp. and *Gymnoascus* spp.) for the biosynthesis of AgNPs. Both strains provided NPs with good physiochemical characteristics however, the PDI for E-AgNPs was low indicating higher homogeneity compared to G-AgNPs which might enhance their antibacterial activity. As an ecofriendly, safe, and cost-effective approach, both myco-synthesized AgNPs could be recommended as antibacterial agents specially against *K. pneumoniae* where their mode of action was reported by TEM and SDS PAGE analysis. However, further investigations are required to validate these findings by increasing the sample size of tested agents and study their antibacterial mechanisms. Overall, this study added new fungal isolates as bio mediators for nanoparticle synthesis with efficiency as antibacterial agents against some human pathogens.

## Data Availability

The datasets generated and/or analyzed during the current study of the two sequences were submitted to GenBank (Accession numbers MN995544.1 and MN995517.1) and are available online in the NCBI repository, persistent in the following web link: https://www.ncbi.nlm.nih.gov/nuccore/MN995544.1?report=genbank, https://www.ncbi.nlm.nih.gov/nuccore/MN995517.
